# Dynamic Changes in Optic Nerve Sheath Diameter on Computed Tomography Predict Decompressive Efficacy and Outcomes in Severe Traumatic Brain Injury

**DOI:** 10.3390/life16050817

**Published:** 2026-05-14

**Authors:** Nicoleta-Larisa Serban, Ioan-Stefan Florian, Teodora Maria Bodog, Matei-Ioan Baltac, Alexandra Cristiana Gache, Daniela Ionescu

**Affiliations:** 1 Department of Neurosurgery, Cluj County Clinical Emergency Hospital, 400347 Cluj-Napoca, Romania; stefanfloriannch@gmail.com (I.-S.F.); (M.-I.B.); 2 Department of Neurosciences, “Iuliu Hatieganu” University of Medicine and Pharmacy, 400012 Cluj-Napoca, Romania; 3 Doctoral School of Biological and Biomedical Sciences, University of Oradea, 410087 Oradea, Romania; 4 Department of Anaesthesia and Intensive Care, County Emergency Hospital Bihor, 410167 Oradea, Romania; 5 Department of Pneumology, Faculty of Medicine, Campus—Corp B, Ovidius University of Constanta, 1 University Alley, 900470 Constanta, Romania; 6 Clinical Hospital of Pneumopthisiology Constanta, 40 Santinelei Street, 900002 Constanta, Romania; 7 Department of Anesthesiology and Intensive Care, “Iuliu Hatieganu” University of Medicine and Pharmacy, 400012 Cluj-Napoca, Romania; 8 Outcome Research Consortium, Cleveland, OH 44195, USA

**Keywords:** traumatic brain injury, optic nerve sheath diameter, decompressive craniectomy, intracranial pressure, computed tomography

## Abstract

Elevated intracranial pressure (ICP) is a major determinant of secondary injury and mortality in severe traumatic brain injury (TBI), yet objective markers of decompressive craniectomy (DC) efficacy remain limited. Optic nerve sheath diameter (ONSD), measurable on computed tomography (CT), has emerged as a non-invasive surrogate of ICP. This study evaluated the relationship between perioperative ONSD changes and clinical and surgical parameters in patients undergoing DC. In this retrospective cohort study, 72 patients with severe TBI were included. ONSD was measured on preoperative and early postoperative CT, and the change (ΔONSD) was analyzed in relation to craniectomy surface area and outcomes. DC resulted in a significant reduction in ONSD (6.44 ± 0.88 mm vs. 5.55 ± 0.82 mm, *p* < 0.001). Larger craniectomy surface areas were associated with greater ΔONSD change (r = −0.31, *p* = 0.008). ΔONSD was independently associated with in-hospital mortality (OR = 0.12, *p* = 0.005), with larger reductions associated with improved survival. Additionally, ΔONSD was correlated with shorter hospital stay (ρ = −0.32, *p* = 0.007). These findings support ΔONSD as a practical imaging biomarker reflecting the physiological response to DC in severe TBI.

## 1. Introduction

Severe traumatic brain injury (TBI) remains one of the leading causes of death and disability in the world [1]. The main contributing factor to mortality and disability from TBI is secondary brain injury associated with intracranial hypertension [2]. Increased intracranial pressure (ICP) lowers cerebral perfusion and worsens ischemic injury and is highly predictive of poor neurological outcome [3,4]. While invasive ICP monitoring is standard of care for detecting intracranial hypertension, it carries procedural risks and is not universally available, especially in low-resource settings [5,6]. An external ventricular drainage (EVD) can be used as an alternative, but increased ICP‚ decreased ventricular size‚ and the existence of a midline shift (MLS) could make its placement difficult [7,8]. Reliable‚ non-intrusive markers of changes in intracranial pressure would have considerable clinical value in these conditions.

The optic nerve sheath diameter (ONSD) has been proposed as a surrogate marker of intracranial pressure (ICP), owing to the anatomical continuity between the optic nerve sheath and the intracranial subarachnoid space, which enables it to reflect changes in cerebrospinal fluid (CSF) pressure [2,5,9,10]. Several studies and meta-analyses have shown an association between increased ONSD and the likelihood of elevated ICP in patients with severe TBI, with consistently high pooled sensitivities (>90%) for detecting intracranial hypertension [2,11]. In acute trauma‚ measurement of the ONSD on CT has the advantage of possibly being done retrospectively from routine scans‚ with no extra cost or effort. In trauma‚ it has been suggested that values between 5.8 and 6.5 mm are diagnostic of ICP [5,9,12]. Although ONSD is assessed using ultrasound in many studies, measurement by CT scan is equally valid [13]. The advantage of the CT examination is that it can be used for both initial assessment and follow-up evaluations and does not require a learning curve, specialized skills, or additional equipment.

While the evidence supporting an association between ONSD and increased ICP continues to grow, the prognostic value of ONSD remains uncertain, especially in patients undergoing a decompressive craniectomy (DC). DC is a well-established treatment for refractory intracranial hypertension, with the potential to markedly reduce ICP and improve survival in carefully selected patients [3]. In contrast‚ radiological markers used to assess decompression effectiveness, such as midline shift or cisternal effacement‚ are a measure of an anatomical change rather than a true measure of change in pressure or volume. Furthermore‚ other studies have reported conflicting results between ONSD values on admission and outcomes in patients who underwent DC‚ suggesting that static ONSD does not correlate accurately with the physiological response to decompression [14,15]. Research suggests that changes in the ONSD‚ rather than preoperative ONSD‚ could be useful for tracking the efficacy of surgical decompression and resolution of increased intracranial pressure. However, there is limited data in the literature on the correlation between postoperative ONSD‚ extent of craniectomy‚ and clinical outcomes.

The present study intended to determine whether ONSD changes on CT scan after a decompressive craniectomy are associated and can be used as an independent predictor of survival in patients with TBI‚ and whether (a) there is a significant decrease in ONSD after decompressive craniectomy‚ (b) whether the magnitude of ONSD decrease correlates with the craniectomy surface area, (c) if ONSD dynamics (ΔONSD) predict survival or correlate with the imaging signs of post-traumatic hydrocephalus (PTH), and (d) can ONSD serve as an objective radiological criterion for the indication of a ventriculo-peritoneal shunt?

## 2. Materials and Methods

### 2.1. Study Design and Patient Selection

The present retrospective‚ observational study has been conducted according to institutional and national regulations on the conduct of human research‚ and the study protocol was approved by the Institutional Ethics Committee of Cluj County Emergency Hospital (No. 1032/61/13 January 2021).

Population. Consecutive adult patients (≥18 years), with no predefined upper age limit, with severe traumatic brain injury (defined as admission with a GCS ≤ 8) who underwent decompressive craniectomy for medically refractory intracranial hypertension were included in this analysis, in line with contemporaneous management for severe TBI [3]. This study took place between January 2019 and December 2023.

Inclusion criteria were:

• Severe TBI requiring decompressive craniectomy;
• Non-contrast head CT that could be used to measure ONSD accurately;
• Availability of an early postoperative CT suitable for determining craniectomy surface area;
• Complete clinical outcome data.

Exclusion criteria included:

• Penetrating brain injury;
• Pre-existing hydrocephalus or prior CSF diversion;
• Severe orbital pathology obscuring ONSD measurement;
• CT studies that were not of adequate quality;
• Key outcome data were missing.

### 2.2. Imaging Protocol

All patients underwent non-contrast head CT imaging on a GE Optima CT660 multidetector CT scanner (GE Healthcare‚ Milwaukee‚ WI‚ USA) according to our institutional neurotrauma imaging protocol.

CT acquisition was performed using the CT parameters used in the acute workup of a patient with severe TBI. Imaging values:

• Raw acquisition slice thickness: 0.6–1.25 mm.
• Axial reconstruction thickness: 3 to 5 mm.
• Standard brain window settings (soft tissue kernel).

Thin-slice datasets (0.6–1.25 mm) were available for image reconstruction and were preferentially used because higher spatial resolution was required to measure the ONSD.

All measurements were performed with axial images. Multiplanar reconstructions (MPR) were not routinely necessary‚ since the optic nerve tract and midline structures could usually be visualized adequately on the axial plane in most cases.

Timing of Imaging

Two timepoints were included in the analysis:

1. Admission CT

The first CT scan was performed on presentation to the emergency department or before neurosurgical intervention. This scan was used for:

• Baseline ONSD measurement;
• Midline shift assessment;
• Marshall CT classification;
• Evaluation of intracranial lesions.

2. Early Postoperative CT

Initial CT scans were performed postoperatively at a mean interval of 24 h after decompressive craniectomy. A follow-up CT‚ at 24 h‚ is conducted as a routine part of postoperative care in our institution. The early postoperative CT was used for:

• Reassessment of ONSD;
• Measurement of craniectomy surface area;
• Postoperative midline shift evaluation;
• Detection of early complications (e.g., rebleeding, hygroma, edema progression).

### 2.3. Optic Nerve Sheath Diameter Measurement

Optic nerve sheath diameter was measured on admission and on the early postoperative CT scan using a standardized CT-based technique [16].

All measurements were made on axial CT images‚ using a dedicated DICOMViewer application (version 1.0.0.105, PixelData) and a set of digital electronic calipers.

We measured the optic nerve sheath 3 mm behind the posterior pole of the globe, perpendicular to the long axis of the optic nerve. We chose this level as other authors have demonstrated that this is the level at which the optic nerve sheath is maximally distensible‚ resulting in a more sensitive parameter for intracranial pressure changes with a higher interobserver agreement.

The measurements were made from the outer-to-outer diameter of the optic nerve sheath.

The following reproducible workflow was applied for each measurement:

1. The globe was identified on axial CT images.
2. The optic nerve was traced posteriorly along its visible course.
3. A point located 3 mm posterior to the posterior scleral margin was identified.
4. At this level, the optic nerve sheath diameter was measured perpendicular to the optic nerve axis.
5. The procedure was repeated for the contralateral eye.

If the angle of the optic nerve was not markedly oblique‚ slice selection was performed by identifying the most representative axial section. ONSD was measured bilaterally in each patient.

**Figure 1:**
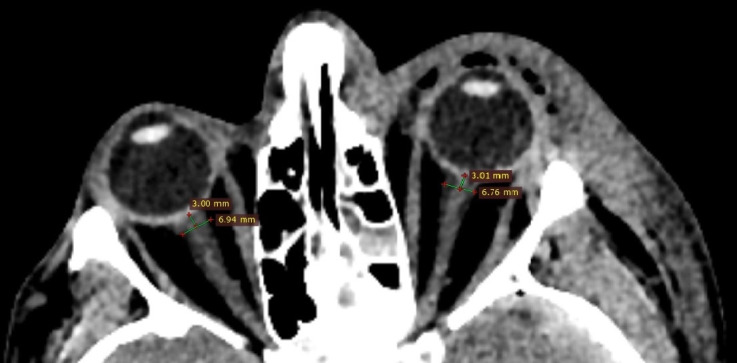
Measurement of optic nerve sheath diameter on axial CT image. The optic nerve sheath diameter is measured 3 mm posterior to the globe, perpendicular to the long axis of the optic nerve, using electronic calipers. The outer-to-outer diameter is recorded. Bilateral measurements are performed, and the mean value is used for analysis.

A schematic illustration of the measurement technique is provided in Figure 1.

The primary metric used in all statistical analyses was

Mean ONSD = (ONSD_right + ONSD_left)/2

The mean bilateral ONSD was chosen to reduce measurement variability and account for potential asymmetry.

Postoperative ONSD was measured using the same technique‚ with the same software settings‚ ensuring internal methodological consistency.

The dynamic variable of interest was defined as

ΔONSD = ONSDpreoperative − ONSDpostoperative

Therefore‚ positive ΔONSD values represented a decrease in the optic nerve sheath diameter postoperatively and were used as a proxy for reduced intracranial pressure after decompressive craniectomy.

All measurements were performed by two neurosurgeons (N.L.S., M.I.B.), and whenever a 10% measurement difference was encountered, a third evaluation was performed by a third neurosurgeon (I.S.F.).

To minimize measurement bias:

• Outcomes were not known to the evaluators.
• Assessors of ONSD were blinded to postoperative craniectomy surface area measurements.
• Admission and postoperative scans were performed using identical technical parameters.

### 2.4. Delayed Postoperative CT

For patients who developed PTH during the postoperative period‚ delayed CT examinations (over 1–2 weeks) were studied to observe the change in ONSD at different times.

Delayed CTs were performed in the presence of clinical and radiological suspicion of hydrocephalus, which was defined as neurological deterioration after an initial period of stability‚ failure to clinically improve as expected in the absence of alternative diagnoses, or worsening of ventriculomegaly on repeat imaging. Neurocritical care practice was reflected by no mandated timeframe for performing CT imaging.

ONSD measurements on delayed CT scans were performed using the same standardized technique described above.

For patients diagnosed with PTH who subsequently underwent ventriculo-peritoneal shunt (VPS) placement, ONSD was measured at three additional predefined timepoints:

• Delayed (PTH) CT, corresponding to the time of hydrocephalus diagnosis.
• Pre-shunt CT, performed immediately before VPS insertion.
• Post-shunt CT, performed after VPS placement as part of routine postoperative assessment.

This made it possible to conduct a complete clinical evaluation of ONSD dynamics:

1. Immediate postoperative period after decompressive craniectomy.
2. Delayed phase associated with hydrocephalus development.
3. Post-intervention phase following CSF diversion.

Only CTs performed in a homogeneous early postoperative time window of 24–48 h after VPS placement were included to ensure temporal consistency.

The following dynamic variables were defined:

• ΔONSD_PTH = ONSD_PTH − ONSD_early postoperative
• ΔONSD_VPS = ONSD_pre-VPS − ONSD_post-VPS

An increased ONSD in the delayed phase of the CT was interpreted as a surrogate marker of an increased ICP with hydrocephalus‚ while ONSD reduction after VPS placement was interpreted as indirect evidence of efficient CSF diversion.

These dynamic measurements were used to investigate relationships between ONSD evolution‚ post-traumatic hydrocephalus development, and the response to cerebrospinal fluid diversion.

### 2.5. Decompressive Craniectomy Surface Area Assessment

The extent of bony decompression was quantified on early postoperative CT scans using a standardized geometric estimation method identical to that previously described in a prior publication [17]. This approach was selected to ensure methodological consistency and allow reproducible comparison between patients.

On the CT performed postoperatively after DC and in the axial view‚ the cranial defect corresponding to the amount of removed bone flap was located. To quantify the area of the craniectomy‚ the axial cut with the maximal area of craniectomy was used. The axial cut was chosen such that the anterior–posterior and cranio-caudal dimensions of the defect were maximally visible and least susceptible to distortion due to the curvature of the skull and obliqueness of the slice.

Maximal orthogonal diameters including maximal anterior–posterior (AP) diameter and maximal craniocaudal (CC) diameter were measured from the bony defect by digital calipers in the same DICOM viewing software used for the ONSD measurement to keep the measurement environment constant for all diameters being assessed. In an attempt to account for the anatomical curvature‚ we used the contralateral (intact) calvarium to estimate the normal curvature of the cranial vault and to avoid systematic underestimation of the decompressed area.

The surface estimate (SE) of decompression was calculated using an elliptical approximation formula:

SE = AP2 × CC2 × π

where AP and CC were expressed in centimeters and π was approximated as 3.1416. This simplified geometric approach was chosen to ensure reproducibility and consistency across patients in a retrospective setting, where standardized three-dimensional reconstructions were not uniformly available.

All measurements were performed by two neurosurgeons (N.L.S., M.I.B.), and whenever a 10% measurement difference was encountered, a third evaluation was done by a third neurosurgeon (I.S.F).

### 2.6. Midline Shift Measurement

The midline shift (MLS) was measured using a similar standardized radiological technique on admission CT scan (preoperatively) and on the early postoperative CT scan (approximately 24 h postoperative). To evaluate structural decompression dynamics, the change in midline shift (ΔMLS) was calculated as

ΔMLS = MLSpreoperative − MLS postoperative

### 2.7. Clinical and Outcome Variables

Demographic and clinical variables were retrospectively retrieved from the institutional electronic medical record and the ICU database‚ including age at hospital admission‚ sex‚ admission GCS score‚ days of mechanical ventilation‚ days of vasopressor treatment, and length of stay in hospital in days (LOS‚ days).

Radiological severity on admission was defined using the Marshall CT classification. Secondary outcomes were post-traumatic hydrocephalus (PTH) and length of stay in hospital.

Additional variables, including the duration of mechanical ventilation and vasopressor therapy, were evaluated to characterize systemic critical illness and analyzed for correlations with ONSD.

### 2.8. Statistical Analysis

Statistical analyses were performed using JASP (Version 0.96.0, JASP Team, Amsterdam, The Netherlands). A two-sided *p*-value < 0.05 was considered statistically significant. To assess the homogeneity of the variables, Levene’s test for equality was performed. Depending on the distribution and homogeneity of variances, parametric or non-parametric tests were applied accordingly.

Continuous variables were assessed for normality using the Shapiro–Wilk test and inspection of distribution plots. Normally distributed continuous variables were described by mean ± standard deviation (SD), while non-normally distributed variables were compared by non-parametric tests. Missing data were negligible and did not impact primary endpoints; therefore‚ a complete-case analysis was performed.

Comparisons between ONSD preoperatively and postoperatively‚ between ONSD pre-VPS insertion and post-VPS insertion when PTH developed‚ were calculated using the Wilcoxon signed-rank test (or paired Student’s *t*-test). The dynamic change in ONSD (ΔONSD)‚ defined as the difference between preoperative and postoperative ONSD‚ was used in all subsequent analyses.

Correlation analyses were performed to assess associations between ΔONSD and continuous clinical and radiological variables. The choice between Pearson’s and Spearman’s correlation coefficients was based on data distribution and measurement characteristics. Pearson’s correlation was applied for normally distributed continuous variables with an assumed linear relationship, while Spearman’s rank correlation was used for non-normally distributed or ordinal variables. For key associations, both coefficients were reported to ensure robustness of findings across parametric and non-parametric assumptions.

Given that the use of Pearson’s correlation implies an underlying linear relationship, additional linear regression analyses were performed for clinically relevant associations (e.g., ΔONSD with craniectomy surface area and length of hospital stay) to further quantify the direction and magnitude of these relationships. Regression assumptions, including linearity and homoscedasticity, were assessed graphically. Scatter plots with fitted regression lines were generated to visually represent these associations.

Comparative analysis between groups (survivors versus nonsurvivors‚ patients with and without post-traumatic hydrocephalus) was performed using the independent samples *t*-test or the Mann–Whitney U test for the mean value of continuous variables and the χ^2^ or Fisher’s exact test for categorical variables.

To determine independent predictors of in-hospital mortality‚ a multivariable logistic regression was performed with in-hospital mortality as the dependent variable. The independent variables included age‚ admission GCS‚ preoperative midline shift‚ craniectomy surface area‚ Marshall CT classification‚ and the change in the ONSD (ΔONSD). Effect sizes were reported using odds ratios (ORs) with 95% confidence intervals (CIs). No external scripts or custom codes were used.

## 3. Results

From a total cohort of 91 patients with TBI who underwent decompressive craniectomy‚ 72 consecutive patients were included in this analysis. They were mostly middle-aged to young‚ with a mean age of 44.2 (standard deviation 19.4‚ range 18–83). The mean admission Glasgow Coma Scale (GCS) score was 3.44 ± 1.18‚ indicating the severity of neurological impairment at presentation.

On average‚ the estimated preoperative midline shift and postoperative midline shift are 11.6 ± 7.4 mm and 8.8 ± 21.5 mm, respectively. The estimated mean craniectomy surface area is 119.4 ± 44.6 cm^2^ (30.0 to 352.9 cm^2^) and‚ like the midline shift‚ the craniectomy surface area sizes vary considerably between patients.

The mean hospital length of stay was 22.4 ± 39.8 days, with a large distribution. There were nine (12.5%) in-hospital deaths. Postoperative hydrocephalus was reported in 10 (11.1%) patients (Table 1).

### 3.1. Effect of Decompressive Craniectomy on Optic Nerve Sheath Diameter

Optic nerve sheath diameter showed a consistent, statistically significant reduction following decompressive craniectomy. The mean preoperative ONSD was 6.44 ± 0.88 mm, whereas the mean postoperative ONSD decreased to 5.55 ± 0.82 mm.

The decrease in ONSD following surgery was significant with Wilcoxon’s signed-rank test (*p* < 0.001)‚ with a large effect size (rank-biserial correlation = 0.87). Consistent results were found using the paired Student *t*-test (t = 9.27‚ *p* < 0.001‚ Cohen’s d = 1.09)‚ indicating a large and clinically meaningful effect.

With a mean ΔONSD of 0.88 mm (±0.74)‚ most patients demonstrated a decrease in ONSD after surgical intervention.

**Figure 2:**
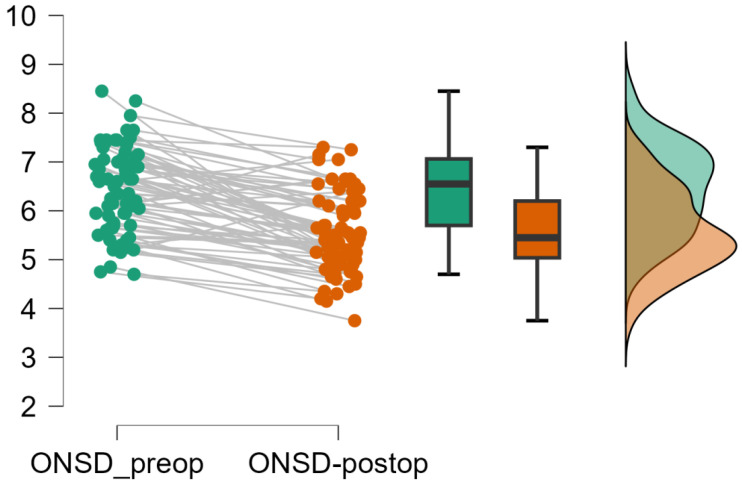
Paired comparison and distribution of preoperative and postoperative optic nerve sheath diameter (ONSD). Individual patient trajectories demonstrate a consistent reduction in ONSD following decompressive craniectomy. Boxplots and density distributions illustrate the overall shift toward lower postoperative values. The decrease was statistically significant (*p* < 0.001).

At an individual level‚ ONSD was reduced after decompressive craniectomy in the majority of cases‚ although there was a minority of cases with either a small reduction in ONSD or no change (Figure 2).

### 3.2. Association Between ΔONSD and Craniectomy Surface Area

The decrease in ONSD was correlated with the surgical decompression. The Pearson correlation analysis showed a moderate negative correlation between the ΔONSD and the size of the craniectomy (r = −0.31‚ *p* = 0.008). This association also held using the non-parametric Spearman rank correlation (ρ = −0.28‚ *p* = 0.016)‚ thus confirming its robustness. A greater craniectomy surface area was associated with a greater postoperative reduction in ONSD.

**Figure 3:**
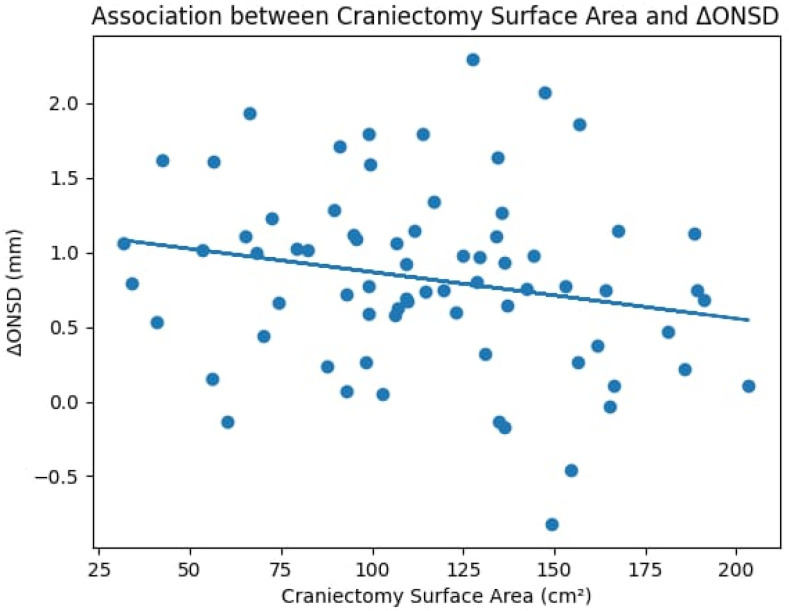
Scatter plot illustrating the relationship between craniectomy surface area and ΔONSD. The fitted regression line demonstrates a significant inverse association, indicating that larger decompressive areas are associated with greater reductions in optic nerve sheath diameter.

To further characterize this relationship, a linear regression analysis was performed, confirming a significant inverse association between craniectomy surface area and ΔONSD (R^2^ = 0.096, *p* = 0.008). The model indicates that larger decompression areas are associated with greater reductions in ONSD. This relationship is illustrated in the corresponding scatter plot with a fitted regression line (Figure 3).

Furthermore‚ the relationship was approximately linear across the range of decompression sizes‚ with no obvious evidence of a discrete surface threshold for the study.

To clarify the clinical relevance of changes in ΔONSD values‚ MDC95 (minimal detectable change at 95% confidence level) of ΔONSD values was calculated based on the observed variability of these measurements (intraclass correlation coefficient was 0.90). The estimated MDC95 of ΔONSD was approximately 0.65 mm.

Changes in ONSD less than this value may be due to variation in measurement‚ while those greater than or equal to 0.65 mm may represent a true decrease in ICP.

Therefore‚ in this light‚ the effects of decompressive craniectomy may actually be better described as a clinically meaningful reduction in ONSD‚ rather than a surface area above a threshold.

No significant association was found between the craniectomy area and either in-hospital mortality (*p* = 0.46) or the degree of postoperative midline shift (*p* = 0.73).

### 3.3. Association Between ΔONSD and Structural Displacement (Midline Shift)

The association between the dynamics of optic nerve sheath diameter and structural brain displacement was assessed.

**Figure 4:**
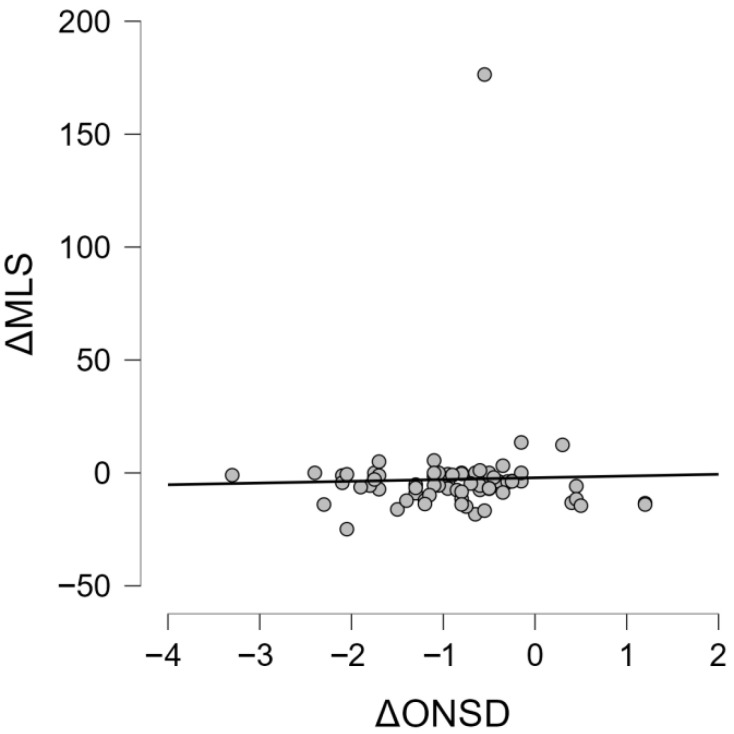
Scatter plot demonstrating no significant association between ΔONSD and change in midline shift (ΔMLS).

Preoperative or postoperative midline shift was not significantly associated with ΔONSD (*p* = 0.68 and *p* = 0.74‚ respectively). There was also no correlation between ΔONSD and ΔMLS determined by a Pearson correlation (r = 0.03; *p* = 0.82) or a Spearman correlation (ρ = −0.04; *p* = 0.74) (Figure 4).

No statistically meaningful effect was found for the reduction in midline shift between preoperative and postoperative scans (*p* = 0.29)‚ despite the reduction in ONSD after decompressive craniectomy.

Postoperative changes in optic nerve sheath diameter occurred independently of macroscopic structural displacement.

### 3.4. Association Between ΔONSD and Clinical Outcomes

Postoperative ONSD dynamics were correlated with clinical outcomes. Univariate analysis found that in-hospital deaths were associated with smaller decreases in ONSD after decompressive craniectomy.

A meaningful inverse correlation between ΔONSD and length of hospitalization (Spearman ρ = −0.32‚ *p* = 0.007) was found. A complementary linear regression analysis was performed to further evaluate the association between ΔONSD and length of hospital stay, confirming the inverse relationship observed in the Spearman correlation. This supports the finding that greater reductions in ONSD are associated with shorter hospitalization duration.

There was no significant correlation between ΔONSD and total duration of mechanical ventilation (*p* = 0.72)‚ duration of vasopressor therapy (*p* = 0.26)‚ or the development of post-traumatic hydrocephalus. A decrease in optic nerve sheath diameter after decompressive craniectomy was associated with survival and recovery parameters.

### 3.5. Multivariable Analysis

To assess whether the variability of ONSD on CT can independently predict mortality outcome‚ a multivariable logistic regression with in-hospital mortality as the dependent variable was used. The independent variables tested were the age‚ admission GCS‚ preoperative midline shift‚ craniectomy surface area (CS)‚ Marshall CT classification‚ and the ΔONSD.

The model was statistically significant (χ^2^ = 29.91‚ *p* < 0.001) and explained 51% of the variance (Nagelkerke R^2^ = 0.51). Of the predictors‚ ΔONSD was considerably associated with in-hospital mortality and was found to be an independent predictor with an odds ratio (OR) of 0.12 (95% CI 0.03 to 0.53‚ *p* = 0.005). A greater drop in the diameter of the optic nerve sheath postoperatively was linked to a lower risk of death.

The other covariates were not found to be independently associated with mortality: age‚ admission GCS‚ preoperative midline shift‚ craniectomy surface area, or Marshall classification. The effect of preoperative midline shift was marginally associated with mortality (*p* = 0.063)‚ but was above the pre-specified level of 0.05 for statistical importance. Detailed regression coefficients and effect estimates are presented in Table 2.

**Figure 5:**
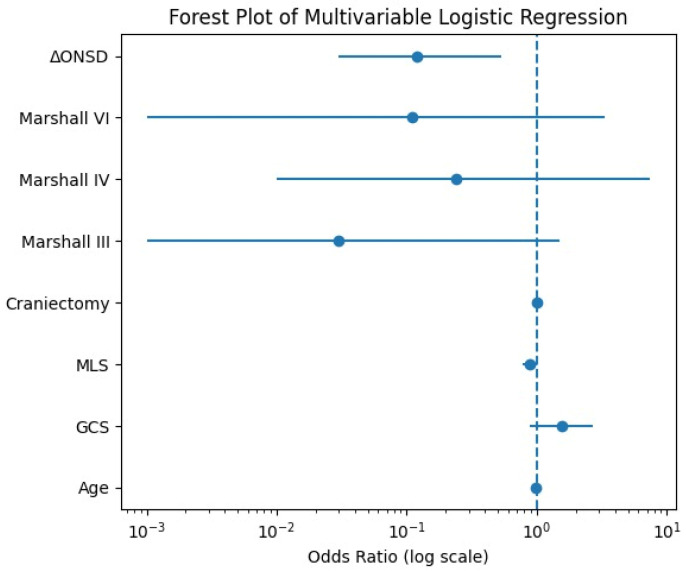
Forest plot of the multivariable logistic regression analysis for in-hospital mortality. Odds ratios (ORs) and 95% confidence intervals are presented for each variable included in the model. The vertical dashed line indicates the reference value (OR = 1).

The results of the multivariable logistic regression analysis are also presented graphically as a forest plot (Figure 5).

### 3.6. Optic Nerve Sheath Diameter Dynamics in Post-Traumatic Hydrocephalus

To further explore the dynamic behavior of ONS beyond the early postoperative phase, additional analyses were performed in patients who developed PTH, as defined in the Section 2.

In 10 patients who underwent delayed CT scans‚ multiple clinically relevant timepoints were selected (the peak phase of hydrocephalus and following VPS placement) for ONSD measurement.

The mean early postoperative ONSD after DC was 5.54 mm‚ rising to a mean of 5.70 mm at the diagnosis of hydrocephalus‚ and decreasing to a mean of 5.06 mm after VPS.

In these patients, the evolution of ONSD followed a biphasic pattern: values decreased after decompressive craniectomy, increased with the development of post-traumatic hydrocephalus, and decreased again following cerebrospinal fluid diversion.

However, no significant linear correlation was observed between ONSD values at the time of hydrocephalus diagnosis (ONSD-PTH) and post-shunt ONSD values (ONSD-VPS) across patients (Pearson r = 0.269, *p* = 0.453; 95% CI −0.434 to 0.768). The coefficient of determination (r^2^ = 0.072) indicates that only a small proportion of the variability in post-shunt ONSD can be explained by ONSD at the time of hydrocephalus.

This apparent discrepancy reflects the difference between intra-individual temporal dynamics and inter-individual variability. While ONSD reliably captures directional changes within the same patient over time, absolute values vary substantially between individuals, limiting the strength of cross-sectional correlations.

## 4. Discussion

Measuring the diameter of the optic nerve sheath to assess intracranial pressure has been well documented in recent years; it is a non-invasive method that can be used both during the initial CT scan upon the patient’s admission and at the patient’s bedside (via ultrasound) [18]. There are also studies attempting to establish a correlation between certain ONSD values and intracranial pressure levels [2,5,19,20], with ONSD values between 6.2 and 6.4 mm correlating with elevated intracranial pressure (≥20 mm Hg) [8,12].

A conceptual summary of the main findings of our study, their physiological interpretation, and potential clinical implications is presented in Table 3.

These findings support ONSD’s role as a dynamic marker of intracranial pressure changes rather than a static anatomical parameter.

Although most published studies use ultrasound to measure ONSD [18,19,21,22,23], citing the fact that it can be performed at the patient’s bedside, it can be repeated as often as necessary, and it does not expose the patient to radiation, there are some drawbacks related to the need for additional equipment and a learning curve, which leads to some variability among examiners. Measuring ONSD on CT, however, is straightforward and can be performed both during the initial examination and at any imaging follow-up. The methodology is standardized in most studies, and the results are reproducible. Studies demonstrate a significant difference between ONSD measured by ultrasound and by CT, with the ultrasound measurement typically smaller [9,24]. Despite these differences and the heterogeneity of the studies, there is a strong correlation between the results of non-invasive measurement methods and increased intracranial pressure [13,25,26,27].

In our study, we focused exclusively on the initial CT scan as well as subsequent postoperative scans to assess the impact of decompressive craniectomy on ONSD. Our findings are consistent with those in the literature: the mean preoperative ONSD was 6.44 ± 0.88 mm, whereas the mean postoperative ONSD decreased to 5.55 ± 0. 82 mm (*p* < 0.001), suggesting intracranial pressure over 20 mm Hg, based on published articles [5,15,20].

Regarding the prognostic value of these measurements, the literature is somewhat contradictory. While some authors emphasize that the ONSD score has lower predictive value for mortality and adverse outcomes compared to the Rotterdam scale [14], other studies demonstrate that it has high predictive value. An analysis of 38 patients showed that an ONSD ≥ 7.32 mm was associated with a 4.66-fold increased risk of mortality [8,28]. Another recently published study found that the optic nerve sheath diameter (ONSD)-to-eyeball transverse diameter (ETD) ratio from CT scans had a sensitivity, specificity, positive predictive value, and negative predictive value of 100%, 95.6%, 72.0%, and 100%, respectively, demonstrating an excellent prediction value for raised ICP [29].

To the best of our knowledge, this study is the first to report ΔONSD for PTH, with a calculated mean of 0.88 ± 0.74 mm in our patients. In univariate analysis, ΔONSD was significantly associated with in-hospital mortality. Patients with smaller postoperative reductions in ONSD were more likely to die during the index hospitalization. A significant inverse correlation was also observed between ΔONSD and length of hospital stay (Spearman ρ = −0.32, *p* = 0.007), indicating that a greater postoperative reduction in sheath diameter was associated with a shorter hospital stay. In multivariate analysis, ΔONSD emerged as the only independent variable significantly associated with in-hospital mortality (OR = 0.12, 95% CI 0.03–0.53, *p* = 0.005). A greater postoperative reduction in optic nerve sheath diameter was associated with a significantly lower probability of death.

Another important aspect, less studied in the literature but highlighted by our study, concerns the lack of correlation between the reduction in ONSD and the size of the craniectomy. Although the most recent recommendations emphasize the need for a large craniectomy with dimensions of 12 × 15 cm and an area of 141.3 cm^2^ [30], this size is not reached in every case‚ and no discrete surface threshold could be identified among our cohort. This suggests a gradual association between decompression and the level of intracranial pressure.

To better characterize the implications of these shifts‚ the MDC95 of the ΔONSD was calculated. This value was found to be 0.65 mm‚ indicating that variations below this threshold could be attributed to variability rather than physiological changes‚ while those above may reflect true physiological alterations.

From this perspective‚ one could argue that a clinically relevant decrease in ONSD is a more appropriate definition of decompressive craniectomy adequacy than a predetermined craniectomy surface area.

Further studies are needed to determine whether a specific threshold for surgical intervention can be identified using dynamic physiological markers, including ONSD.

Finally, the role of ONSD as a predictive factor for the development of post-traumatic hydrocephalus and as an objective criterion for indicating DVP must be emphasized. Few studies in the literature correlate ONSD with the development of communicating or non-communicating hydrocephalus. Lee and colleagues published a study on 64 patients with hydrocephalus of various etiologies, demonstrating that ONSD was linearly correlated with ICP in adults with both communicating and non-communicating hydrocephalus and was a predictor of increased ICP with good discriminatory power [16].

Hall et al. [21] demonstrated via ultrasound that ONSD is higher in children with drainage dysfunction than in those with normal drainage function. Using CT assessment of ONSD, Zaidi et al. [31] demonstrated increased ONSD in nine children with shunt malfunction.

In addition to these immediate postoperative changes in decompressive craniectomy‚ our study has shown that the optic nerve sheath diameter changes with time‚ especially in post-traumatic hydrocephalus. The increase in ONSD while developing PTH‚ followed by the decrease in size following ventriculo-peritoneal shunt placement‚ suggests that this measurement does accurately reflect real-time changes in ICP status.

The fact that there is no significant linear correlation between ONSD values in hydrocephalus and after CSF diversion suggests that ONSD measurements should not be interpreted as an absolute value‚ but as one that should be evaluated in the specific clinical context. From this perspective‚ ONSD could be considered an objective indication for VPS in patients with severe TBI in poor neurological condition‚ since these patients have no clinical and radiological criteria for following its change in dynamics for each patient.

From a clinical perspective, the interpretation of ONSD remains challenging, particularly in the absence of universally accepted threshold values applicable across heterogeneous patient populations. In the present study, we deliberately focused on dynamic changes in ONSD (ΔONSD) rather than absolute values, as inter-individual variability may limit the clinical utility of fixed cutoffs.

Our findings suggest that ΔONSD may serve as a practical, non-invasive marker of the physiological response to decompressive craniectomy. A greater postoperative reduction in ONSD was consistently associated with improved clinical outcomes, including lower in-hospital mortality and shorter length of stay.

To enhance clinical interpretability, we introduced the concept of the minimal detectable change at the 95% confidence level (MDC95 ≈ 0.65 mm), which provides a pragmatic threshold for distinguishing true physiological variation from measurement variability. From a clinical standpoint, ΔONSD values exceeding this threshold may indicate a meaningful response to surgical decompression, whereas smaller changes should be interpreted with caution.

Importantly, these findings support a dynamic, patient-centered approach to ONSD interpretation, in which relative changes over time may provide more clinically relevant information than isolated measurements. In this context, ONSD may complement traditional radiological markers and contribute to a more comprehensive assessment of intracranial pressure dynamics. These clinical implications are summarized in Table 3.

Nevertheless, the absence of direct intracranial pressure measurements in the present study precludes the definition of validated clinical cutoff values. Prospective studies integrating serial imaging and invasive monitoring are warranted to establish standardized thresholds for clinical decision-making.

## 5. Limitations

The limitations of our study include its retrospective design, variability in CT acquisition across referring centers, and differences in slice thickness, which may have influenced ONSD measurements.

A further important limitation relates to ICP monitoring. Although invasive ICP measurements were available in a subset of patients, these data were not systematically collected across the entire cohort and were therefore not suitable for inclusion in the statistical analysis. Consequently, a direct physiological validation of ONSD dynamics against absolute ICP values could not be consistently performed.

Although ONSD has been extensively validated in prior studies as a non-invasive surrogate marker of intracranial pressure, our findings should be interpreted within this context.

Importantly, this study does not aim to establish ONSD as a quantitative substitute for invasive ICP monitoring, but rather to evaluate whether dynamic changes in ONSD (ΔONSD) reflect relative physiological responses to decompressive craniectomy. In this regard, intra-patient changes may provide meaningful information even in the absence of absolute ICP calibration.

Therefore, ΔONSD should be regarded as an indirect imaging biomarker, complementary to clinical and radiological parameters, rather than a replacement for invasive ICP monitoring. Future prospective studies incorporating simultaneous ICP measurements are needed to validate this relationship further.

Another important limitation relates to the timing of postoperative imaging. In this study, early postoperative CT scans were performed at approximately 24 h after decompressive craniectomy, in accordance with institutional protocol. While this approach ensures consistency across patients, it may not fully capture the immediate intracranial pressure dynamics occurring in the early postoperative period.

Rapid changes in intracranial pressure following surgical decompression, as well as early secondary deterioration, may occur within the first hours after surgery and therefore may not be reflected in the measured ONSD values. As such, the observed ΔONSD likely represents an early but not immediate physiological response to decompression.

This temporal limitation should be considered when interpreting our findings, and future studies incorporating earlier or serial imaging assessments could provide a more comprehensive understanding of ONSD dynamics.

Another methodological limitation relates to the estimation of craniectomy surface area. In this study, the extent of decompression was approximated using an elliptical formula based on measurements obtained from a single axial CT slice. While this approach provides a practical and reproducible estimation, it does not fully account for the complex three-dimensional geometry of the cranial defect and may introduce measurement error.

Although efforts were made to standardize measurements by selecting the axial slice with the maximal defect dimensions and by using independent evaluators, some degree of imprecision is inherent in this method. However, as our analyses focused on relative differences between patients rather than precise volumetric quantification, this limitation is unlikely to have substantially influenced the observed associations.

Future studies incorporating three-dimensional reconstruction techniques or volumetric analysis could provide a more accurate assessment of the extent of decompression and further refine these findings.

## 6. Conclusions

We conclude that ONSD measured by CT scan is an early and clinically useful indicator for ICP changes in patients with severe TBI undergoing decompressive craniectomy.

A meaningful postoperative decrease in ONSD after surgery suggests that surgical decompression in these patients is successful. The degree of reduction in ONSD (ΔONSD) is an independent predictor of survival and hospital discharge and thus may represent a significant prognostic biomarker in this context.

It has been suggested that dynamic changes in ONSD rather than static radiological parameters better represent normalization of intracranial pressure and therapeutic response.

Furthermore‚ the transient nature of ONSD changes in post-traumatic hydrocephalus supports its value as a real-time imaging biomarker of rapid fluctuations in intracranial pressure.

Overall‚ ΔONSD may represent a simple‚ non-intrusive‚ and widely available tool for evaluating decompressive efficacy and guiding clinical decision-making in severe traumatic brain injury.

## Figures and Tables

**Table 1:** Baseline characteristics of the study cohort.

Variable	Value
Age (years), mean ± SD	44.2 ± 19.4
Admission GCS, mean ± SD	3.44 ± 1.18
Preoperative ONSD (mm)	6.44 ± 0.88
Postoperative ONSD (mm)	5.55 ± 0.82
ΔONSD (mm)	0.88 ± 0.74
ONSD at PTH diagnosis (mm)	5.70 ± 0.47
Post-VPS ONSD (mm)	5.05 ± 0.35
Preoperative MLS (mm)	11.6 ± 7.4
Postoperative MLS (mm)	8.8 ± 21.5
Craniectomy surface (cm^2^)	119.4 ± 44.6
Hospital stay (days)	22.4 ± 39.8
Mortality, *n* (%)	9 (12.5%)
Post-traumatic hydrocephalus, *n* (%)	10 (11.1%)

Values marked with () were calculated in the subgroup of patients with post-traumatic hydrocephalus (*n* = 10).

**Table 2:** Multivariable logistic regression analysis identifying independent predictors of in-hospital mortality in patients undergoing decompressive craniectomy for severe traumatic brain injury.

Variable	OR	95% CI	*p*-Value
Age	0.98	0.94–1.02	0.344
Admission GCS	1.55	0.89–2.69	0.124
Preoperative midline shift	0.88	0.78–1.01	0.063
Craniectomy surface area	1.00	0.98–1.02	0.947
Marshall grade III	0.03	0.00–1.50	0.077
Marshall grade IV	0.24	0.01–7.43	0.415
Marshall grade VI	0.11	0.00–3.32	0.205
ΔONSD	0.12	0.03–0.53	0.005

Odds ratios (ORs) with 95% confidence intervals (CIs) are presented. ΔONSD represents the difference between preoperative and postoperative optic nerve sheath diameter (ONSD). The model was statistically significant (χ^2^ = 29.91, *p* < 0.001), with a Nagelkerke R^2^ of 0.51. A two-sided *p*-value < 0.05 was considered statistically significant.

**Table 3:** Conceptual summary of main findings and clinical implications.

Finding	Statistical Result	Interpretation	Clinical Implication
Significant reduction in ONSD after decompressive craniectomy	6.44 ± 0.88 mm vs. 5.55 ± 0.82 mm, *p* < 0.001	Reflects reduction in intracranial pressure following surgical decompression	Supports ONSD as a non-invasive marker of decompressive efficacy
ΔONSD correlated with craniectomy surface area	r = −0.31, *p* = 0.008	Larger decompressive surface associated with greater ICP reduction	Suggests surgical extent influences physiological response, although no clear threshold identified
ΔONSD independently associated with in-hospital mortality	OR = 0.12, *p* = 0.005	Greater postoperative decrease in ONSD associated with improved survival	ΔONSD may serve as a prognostic biomarker
ΔONSD correlated with hospital length of stay	ρ = −0.32, *p* = 0.007	Greater reduction in ONSD associated with faster recovery	May reflect early favorable clinical evolution
No association between ΔONSD and ΔMLS	*p* > 0.7	ONSD reflects pressure dynamics rather than structural displacement	Complements traditional imaging markers (e.g., midline shift)
Dynamic changes in ONSD in post-traumatic hydrocephalus	Increase at PTH, decrease after VPS	ONSD reflects dynamic CSF pressure changes	Potential role in monitoring hydrocephalus and response to CSF diversion
Minimal detectable change (MDC95) for ΔONSD ≈ 0.65 mm	Derived from measurement variability	Threshold distinguishing true physiological change from measurement error	Practical reference for interpreting clinically meaningful ONSD variation

Abbreviations: ONSD—optic nerve sheath diameter; ΔONSD—change in ONSD; MLS—midline shift; PTH—post-traumatic hydrocephalus; VPS—ventriculo-peritoneal shunt.

## Data Availability

The datasets generated and analyzed in the current study are not publicly available due to ethical restrictions and patient confidentiality, but are available from the corresponding author upon reasonable request.
